# DRL-Based Beam Split Alleviation for Movable Antenna-Enabled Near-Field Wideband Communications

**DOI:** 10.3390/s26103172

**Published:** 2026-05-17

**Authors:** Tingting Zhang, Rui Jiang, Haibo Dai, Changpeng Zhou, Youyun Xu

**Affiliations:** 1 School of Communication and Information Engineering, Nanjing University of Posts and Telecommunications, Nanjing 210023, China; 2022010309@njupt.edu.cn (T.Z.); j_ray@njupt.edu.cn (R.J.); 2022010310@njupt.edu.cn (C.Z.); 2 School of Internet of Things, Nanjing University of Posts and Telecommunications, Nanjing 210023, China; hbdai@njupt.edu.cn

**Keywords:** deep reinforcement learning, movable antennas, near-field wideband communication, beam split effect, soft actor–critic

## Abstract

Near-field communication is regarded as a key enabling technology for future 6G wireless systems. However, when operating over wide bandwidths, the beam split effect arising from frequency-independent analog phase shifters leads to significant beamforming gain degradation. Different from existing works that address this issue through true-time-delay hardware, this paper exploits the emerging movable antenna technology for beam split alleviation. Specifically, we consider a movable antenna-enabled near-field wideband uplink system with an analog beamforming architecture. Under this setup, we jointly optimize the analog phase shifts and antenna positions to maximize the minimum beamforming gain across all subcarriers. The formulated problem is highly non-convex due to the constant-modulus constraint on the analog combiner and the nonlinear dependence of the near-field channel on antenna positions, which makes conventional optimization methods difficult to apply. To this end, we develop a deep reinforcement learning framework based on the soft actor–critic algorithm that operates in a continuous action space and effectively handles the non-smooth max-min objective. Simulation results show that the proposed approach alleviates the beam split effect and achieves a higher minimum beamforming gain than conventional schemes.

## 1. Introduction

Extremely large-scale antenna arrays operating at millimeter-wave and terahertz bands are envisioned as a cornerstone of future sixth-generation (6G) wireless systems [1,2]. The combination of large array apertures and high carrier frequencies extends the near-field region to practical communication distances, where the spherical wavefront structure enables beam focusing at specific spatial locations rather than merely angular steering [3]. However, when such systems operate over wide bandwidths, the frequency-independent nature of analog phase shifters causes different subcarriers to experience distinct beam focal points, a phenomenon known as the beam split effect, which results in severe beamforming gain loss at edge subcarriers [4]. To address this issue, true-time-delay (TTD)-based hardware architectures have been proposed to provide frequency-dependent phase shifts [5,6]. In particular, an adaptive TTD configuration with a switch network is developed in [7], where an unsupervised transformer is employed to jointly optimize the hybrid beamformer and the TTD-phase shifter connection for arbitrary user locations and array shapes. In [8], a Riemannian optimization framework is proposed for fully connected TTD-based hybrid beamforming in multi-user near-field settings. However, TTD-based approaches generally require additional hardware components (e.g., delay lines, switch networks) that increase the system cost and power consumption, especially for large arrays.

As an alternative to TTD, movable antenna (MA) technology has recently attracted growing attention as a promising paradigm that introduces additional spatial degrees of freedom by allowing antenna elements to adjust their physical positions within a confined region [9,10]. For instance, the energy efficiency of MA-aided multi-user uplink systems is investigated in [11], where antenna movement delay and energy consumption are jointly accounted for in the optimization. In [12], MAs are applied to integrated sensing and communication systems, where antenna positions and beamforming coefficients are jointly optimized to enhance both communication rate and sensing mutual information. Beyond these far-field studies, MA has also shown great potential in near-field communication scenarios. For example, the authors in [13] consider MA-enabled near-field multiuser communication and jointly optimize antenna positions and beamforming to maximize the minimum achievable rate. In [14], MAs are equipped at both the base station and users to minimize the transmit power for multiuser downlink communication. Furthermore, MA is exploited for physical layer security in [15], where hybrid beamformers and MA positions are jointly designed for secrecy rate maximization in near-field multiple-input multiple-output (MIMO) systems. The work in [16] extends MA to near-field integrated sensing and communication (ISAC), jointly optimizing beamformers and antenna positions to maximize a weighted sum of sensing and communication rates. In addition, a deep learning framework is developed in [17] for multibeamforming design and antenna position optimization in extremely large-scale MIMO systems. However, the above works all focus on narrowband near-field systems. In wideband scenarios, MA can further serve as a means to alleviate the beam split effect, since physically adjusting antenna positions modifies propagation path lengths and thereby partially compensates for frequency-dependent phase misalignment that phase-only beamforming cannot correct. Along this line, the authors in [18] formulate a max-min beamforming gain optimization problem for a wideband near-field system, where the antenna positions are optimized under a fully digital architecture via a smoothed gradient-descent-ascent method. In [19], a flexible intelligent metasurface is proposed to mitigate beam squint by optimizing the physical deformation of the antenna surface to minimize the total array gain loss across the bandwidth. It is worth noting that the work in [18] considers a fully digital architecture with per-antenna RF chains, and the study [19] requires specialized deformable hardware, neither of which can be directly applied to analog beamforming architectures that are more desirable in extremely large-scale antenna systems due to the significantly reduced hardware cost and power consumption. To date, how to exploit MA for beam split alleviation under analog beamforming architectures, which employ only frequency-independent phase shifters, remains an open problem.

To fill this gap, this paper considers a movable antenna-enabled near-field wideband uplink system with an analog beamforming architecture. We formulate a max-min optimization problem that jointly optimizes the analog phase shifts and antenna positions to maximize the minimum beamforming gain across all subcarriers. The formulated problem is highly non-convex due to the non-smooth max-min objective, the constant-modulus constraint on the analog combiner, and the nonlinear dependence of the near-field channel on antenna positions. Conventional techniques such as alternating optimization and gradient-based methods are not effective for this problem. To this end, we resort to deep reinforcement learning (DRL), which has demonstrated strong capability in solving complex beamforming optimization problems in far-field systems [20,21]. More recently, learning-based methods have been extended to near-field wideband scenarios. For instance, a deep learning framework is proposed in [22] to learn beam focusing with hybrid TTD-phase shifter architectures for wideband massive MIMO, though with fixed antenna positions. DRL has also been applied to joint beamforming and antenna position design for MA systems, but under fully digital architectures [23]. Nevertheless, none of the existing works address the joint optimization of MA positions and analog beamforming for beam split alleviation in near-field wideband systems. Motivated by this, we develop a soft actor–critic (SAC)-based DRL framework that jointly optimizes antenna positions and analog phase shifts in a continuous action space. Compared to gradient-based methods, SAC operates in a model-free manner, and its entropy regularization encourages exploration, which mitigates the risk of converging to local optima. The non-smooth max-min objective is handled through reward shaping. Simulation results demonstrate that the proposed approach achieves a significantly higher minimum beamforming gain than existing schemes.

The main contributions of this paper are summarized as follows.

- We consider a near-field wideband uplink system where a base station employs a movable antenna array with analog beamforming. A max-min optimization problem is formulated to jointly optimize the analog phase shifts and antenna positions, with the goal of maximizing the minimum beamforming gain across all subcarriers for beam split alleviation.
- We propose an SAC-based DRL framework to solve the formulated problem. In the designed Markov decision process, the phase shifts and antenna positions serve as continuous actions, and the minimum beamforming gain is used as the reward to directly drive the policy towards beam split alleviation. Moreover, the constant-modulus constraint is naturally satisfied by parameterizing the combining vector through phase angles, and the mobility constraint is enforced by clipping the antenna displacements.
- Numerical results show that the proposed approach achieves a higher minimum beamforming gain than fixed phase combining and phase-only optimization, and the gain distribution across subcarriers is significantly more uniform, which confirms the effectiveness of the proposed method in alleviating the beam split effect.

The remainder of this paper is organized as follows. Section 2 presents the system model of the MA-enabled near-field wideband uplink system, and formulates the max-min beamforming gain optimization problem. Section 3 details the proposed SAC-based DRL framework. Simulation results are presented in Section 4, followed by conclusions in Section 5.

Notation: Boldface lower-case and upper-case letters denote vectors and matrices, respectively. ${\left(\cdot \right)}^{T}$ and ${\left(\cdot \right)}^{H}$ denote the transpose and conjugate transpose, respectively. |·| denotes the absolute value, ∠(·) the phase angle, $j=\sqrt{-1}$ the imaginary unit, and E{·} the expectation operator. R denotes the set of real numbers.

## 2. System Model and Problem Formulation

**Figure 1:**
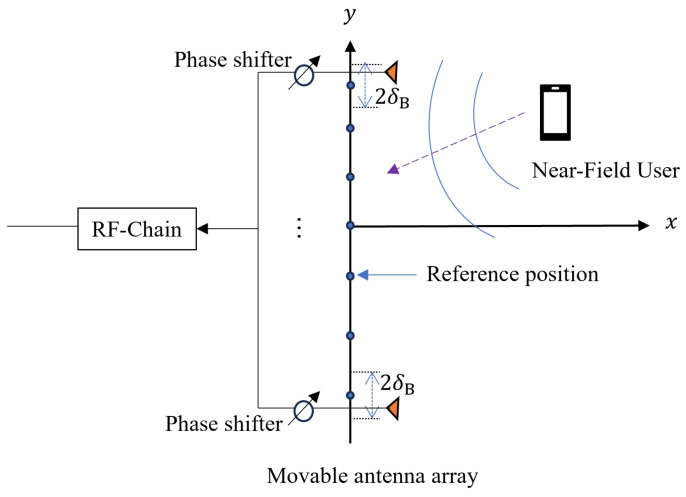
Illustration of MA-enabled near-field wideband uplink systems with analog beamforming architecture.

As illustrated in Figure 1, we consider a near-field wideband uplink system where a single-antenna user communicates with a base station (BS) that is equipped with an *N*-element movable extremely large-scale antenna array. The array elements are aligned along the *y*-axis, and each element is allowed to adjust its position locally around a reference location $y_{m}^{\mathrm{ref}}$. The position of the *m*-th element is given by

(1) $$p_{m}=\left(0,\;y_{m}^{\mathrm{ref}}+\delta_{m}\right),$$

where $\delta_{m}\in \left[-\delta_{B},\delta_{B}\right]$ represents the displacement of the *m*-th element, and $\delta_{B}$ denotes the maximum allowable displacement.

For wideband transmission, the system employs orthogonal frequency division multiplexing with *K* subcarriers, where the subcarrier frequencies ${\left\{f_{k}\right\}}_{k=1}^{K}$ are uniformly distributed over a total bandwidth *B* centered at $f_{c}$. Assuming that the user is located at $p_{u}=\left(x_{u},y_{u}\right)$ within the near-field region of the array, the propagation distance from the user to the *m*-th element can be written as

(2) $$d_{m}\left(\delta_{m}\right)=\sqrt{{\left(y_{u}-y_{m}^{\mathrm{ref}}-\delta_{m}\right)}^{2}+x_{u}^{2}}.$$

As in [13,18], we consider LoS-dominant propagation, which is practical at millimeter-wave frequencies and has been widely adopted in recent near-field MA studies. Under the spherical wavefront assumption, the near-field channel between the user and the *m*-th element at the *k*-th subcarrier is given by [24]

(3) $${\left[h_{k}\left(\delta \right)\right]}_{m}=\frac{\lambda_{k}}{4\pi d_{m}\left(\delta_{m}\right)}\exp\;\left(-j\frac{2\pi }{\lambda_{k}}d_{m}\left(\delta_{m}\right)\right),$$

where $\lambda_{k}=c/f_{k}$ denotes the wavelength at the *k*-th subcarrier with *c* being the speed of light, and $\delta ={\left[\delta_{1},\ldots ,\delta_{N}\right]}^{T}$ collects all displacement variables.

At the BS, the received signals across all elements are combined using a phase-shifter-based analog combiner

(4) $$w\left(\theta \right)={\left[e^{j\theta_{1}},\;e^{j\theta_{2}},\;\ldots ,\;e^{j\theta_{N}}\right]}^{T},$$

where $\theta_{m}\in \left[0,2\pi \right)$ is the phase shift at the *m*-th element and the constant-modulus constraint $|w_{m}|\;=\;1$ holds for all *m*. Correspondingly, the resulting beamforming gain at the *k*-th subcarrier is

(5) $$G_{k}\left(\theta ,\delta \right)={\left|w^{H}\left(\theta \right)h_{k}\left(\delta \right)\right|}^{2}.$$

Since the analog phase shifters are frequency-independent, all subcarriers share the same combining vector w(θ). As the operating frequency deviates from the center, the phase misalignment between w(θ) and $h_{k}\left(\delta \right)$ grows, causing different subcarriers to focus their beam energy at distinct spatial positions. This is the beam split effect, which leads to severe degradation of the beamforming gain at edge subcarriers. To alleviate this effect, we are interested in maximizing the minimum beamforming gain across all subcarriers by jointly optimizing the analog phase shifts θ and the antenna positions δ. This leads to the following max-min optimization problem

(6) $$\begin{array}{cc} \max_{\theta ,\;\delta }\; & \min_{k\in \{1,\ldots ,K\}}\;G_{k}\left(\theta ,\delta \right)\\ s.t.\; & -\delta_{B}\leq \delta_{m}\leq \delta_{B},\;\forall m,\\ & |w_{m}\left(\theta \right)|=1,\;\forall m. \end{array}$$

Problem (6) is highly challenging to solve due to the following reasons. First, the minimum operator in the objective is non-smooth, making gradient-based optimization methods difficult to apply directly. Second, the near-field channel $h_{k}\left(\delta \right)$ depends nonlinearly on the antenna positions through the distance function $d_{m}\left(\delta_{m}\right)$, which is embedded in both the amplitude and phase of the channel coefficients. Third, the constant-modulus constraint on w(θ) further restricts the feasible set. To address these challenges, in the next section, we develop a deep reinforcement learning framework based on the SAC algorithm, which can effectively handle non-smooth objectives and complex constraints through appropriate state-action design and reward shaping.

## 3. Proposed SAC-Based DRL Algorithm

**Figure 2:**
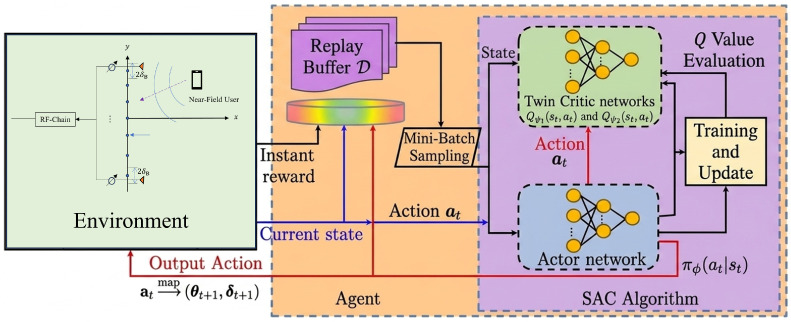
Framework of the proposed SAC-based DRL method for joint phase shift and antenna position optimization.

In this section, we present the proposed DRL-based algorithm for solving Problem (6). The key idea is to model the sequential adjustment of the analog phase shifts θ and antenna positions δ as a Markov decision process (MDP), and train an agent to learn a policy that progressively improves the minimum beamforming gain across subcarriers. We adopt the soft actor–critic (SAC) algorithm due to its ability to handle high-dimensional continuous action spaces and its entropy regularization mechanism that balances exploitation and exploration, which helps avoid local optima. The overall framework is illustrated in Figure 2, where the agent observes the current beamforming configuration, outputs incremental adjustments to both the phase shifts and antenna positions, and receives a reward based on the resulting minimum beamforming gain.

### 3.1. MDP Formulation

To apply DRL, we first cast Problem (6) into an MDP defined by the tuple (S,A,P,r,γ), where S denotes the state space, A the action space, P the transition probability, *r* the reward function, and γ∈(0,1) the discount factor. Each episode consists of *T* interaction steps, during which the agent iteratively refines θ and δ starting from an initial configuration. The design of each MDP element is detailed as follows.

The state at step *t* consists of the current phase shifts, antenna positions, and two gain indicators, i.e.,

(7) $$s_{t}={\left[\theta_{t}^{T},\;\delta_{t}^{T},\;G_{\min,t},\;G_{\mathrm{avg},t}\right]}^{T}\in R^{2N+2},$$

where $G_{\min,t}=\min_{k}G_{k}\left(\theta_{t},\delta_{t}\right)$ and $G_{\mathrm{avg},t}=\frac{1}{K}\sum_{k=1}^{K}G_{k}\left(\theta_{t},\delta_{t}\right)$ denote the minimum and average beamforming gains across all subcarriers, respectively. Including these two scalar features provides the agent with direct feedback on the beam split severity. Specifically, a large gap between $G_{\mathrm{avg},t}$ and $G_{\min,t}$ indicates that the gain is concentrated on a few subcarriers while the edge subcarriers suffer from severe degradation, which signals the agent to adjust the phase shifts and antenna positions accordingly.

The action represents incremental updates to both the phase shifts and antenna positions, given by

(8) $$a_{t}={\left[\Delta \theta_{t}^{T},\;\Delta \delta_{t}^{T}\right]}^{T}\in R^{2N}.$$

After the agent outputs $a_{t}$, the phase shifts and positions are updated as

(9) $$\theta_{t+1}=\left(\theta_{t}+\alpha_{\theta }\Delta \theta_{t}\right)\;\;\;\mathrm{mod}\;2\pi ,$$

(10) $$\delta_{t+1}=\mathrm{clip}\left(\delta_{t}+\alpha_{\delta }\Delta \delta_{t},\;-\delta_{B},\;\delta_{B}\right),$$

where $\alpha_{\theta }$ and $\alpha_{\delta }$ are step-size scaling factors. The modulo operation ensures the phase values remain in [0,2π), and the clipping operation enforces the antenna mobility constraint $|\delta_{m}|\leq \delta_{B}$. Note that the constant-modulus constraint $|w_{m}|=1$ is automatically satisfied since the combining vector is parameterized as $w_{m}=e^{j\theta_{m}}$.

The reward at step *t* is defined to align with the objective function in (6), i.e.,

(11) $$r_{t}=\min_{k=1,\ldots ,K}G_{k}\left(\theta_{t+1},\delta_{t+1}\right),$$

which equals the minimum beamforming gain after the action is applied. This reward directly incentivizes the agent to equalize the gain distribution across subcarriers and improve the performance of the most degraded subcarrier.

### 3.2. SAC Algorithm

Given the MDP formulation above, we employ the SAC algorithm [25] to learn the optimal policy. SAC is an off-policy actor–critic method that maximizes the expected cumulative reward augmented by a policy entropy term

(12) $$\max_{\pi }\;E_{\pi }\left[\sum_{t=0}^{T-1}\gamma^{t}\left(r_{t}+\alpha_{H}H\left(\pi \left(\cdot |s_{t}\right)\right)\right)\right],$$

where $\alpha_{H}>0$ is the temperature parameter that balances reward maximization and entropy H(·) of the policy. The entropy term encourages the agent to explore diverse actions, which is important for escaping local optima in the non-convex landscape of Problem (6).

As shown in Figure 2, the SAC framework consists of three main components: an actor network, twin critic networks, and a replay buffer. The actor network $\pi_{\phi }\left(a_{t}|s_{t}\right)$, parameterized by ϕ, takes the state $s_{t}$ as input and outputs a Gaussian distribution over actions. The mean and log-standard-deviation are produced by two separate output heads, and the sampled action is squashed by a tanh function to ensure boundedness. Two critic networks, $Q_{\psi_{1}}\left(s_{t},a_{t}\right)$ and $Q_{\psi_{2}}\left(s_{t},a_{t}\right)$, independently estimate the expected return given a state-action pair, and the minimum of the two estimates is used to reduce overestimation bias. A replay buffer D stores transition tuples $\left(s_{t},a_{t},r_{t},s_{t+1}\right)$ collected during training, enabling off-policy learning via random mini-batch sampling.

The critic networks are trained by minimizing the soft Bellman residual

(13) $$L\left(\psi_{i}\right)=E_{(s,a,r,s^{\prime })∼D}\left[{\left(Q_{\psi_{i}}\left(s,a\right)-y\right)}^{2}\right],$$

where the target value is $y=r+\gamma \left(\min_{i=1,2}Q_{{\bar{\psi }}_{i}}\left(s^{\prime },{\tilde{a}}^{\prime }\right)-\alpha_{H}\log\pi_{\phi }\left({\tilde{a}}^{\prime }|s^{\prime }\right)\right)$ with ${\tilde{a}}^{\prime }∼\pi_{\phi }\left(\cdot |s^{\prime }\right)$ and ${\bar{\psi }}_{i}$ denoting the parameters of the target critic networks, which are updated via soft tracking: ${\bar{\psi }}_{i}\leftarrow \tau \psi_{i}+\left(1-\tau \right){\bar{\psi }}_{i}$ with τ≪1.

The actor network is updated by minimizing

(14) $$L\left(\phi \right)=E_{s∼D}\left[\alpha_{H}\log\pi_{\phi }\left(\tilde{a}|s\right)-\min_{i=1,2}Q_{\psi_{i}}\left(s,\tilde{a}\right)\right],$$

where $\tilde{a}$ is sampled from $\pi_{\phi }\left(\cdot |s\right)$ using the reparameterization trick to allow gradient backpropagation through the sampling process.

The temperature parameter $\alpha_{H}$ is automatically adjusted by minimizing

(15) $$L\left(\alpha_{H}\right)=E_{\tilde{a}∼\pi_{\phi }}\left[-\alpha_{H}\left(\log\pi_{\phi }\left(\tilde{a}|s\right)+\bar{H}\right)\right],$$

where $\bar{H}=-2N$ is the target entropy set to the negative of the action dimension, following standard practice.

Regarding the network architecture, the actor network takes the state $s_{t}\in R^{2N+2}$ as input, which encodes the current phase shifts, antenna positions, and gain indicators. Since the optimization variables θ and δ interact with the channel in a highly nonlinear manner through the distance function and the exponential phase term in (3), we employ two fully connected hidden layers with rectified linear unit (ReLU) activations to capture these nonlinear mappings, i.e.,

(16) $$z_{1}=\mathrm{ReLU}\left(W_{1}s_{t}+b_{1}\right),\;\;\;z_{2}=\mathrm{ReLU}\left(W_{2}z_{1}+b_{2}\right),$$

where $W_{l}$ and $b_{l}$ are the weight matrix and bias vector of the *l*-th layer. The output layer is split into two heads that produce the mean $\mu_{t}\in R^{2N}$ and the log-standard-deviation $\log\sigma_{t}\in R^{2N}$, respectively, which together parameterize a Gaussian policy $\pi_{\phi }\left(a_{t}|s_{t}\right)=N\left(\mu_{t},\mathrm{diag}\left(\sigma_{t}^{2}\right)\right)$. The mean head determines the direction of the phase and position adjustments, while the standard-deviation head controls the exploration magnitude, allowing the agent to adaptively balance exploitation of known good configurations and exploration of new ones during training. The log-standard-deviation is clamped to $\left[\sigma_{\min},\sigma_{\max}\right]$ to prevent numerical instability. The raw action ${\hat{a}}_{t}$ is sampled from $N(\mu_{t},\mathrm{diag}\left(\sigma_{t}^{2}\right))$ and then squashed by a tanh function to yield the bounded action $a_{t}=\tanh\left({\hat{a}}_{t}\right)\in {\left[-1,1\right]}^{2N}$. The first *N* components correspond to the phase shift updates scaled by $\alpha_{\theta }$, and the remaining *N* components correspond to the position updates scaled by $\alpha_{\delta }$. This split design allows the actor to independently control the two types of optimization variables while sharing the feature extraction layers.

Each critic network takes the concatenation of state and action ${\left[s_{t}^{T},a_{t}^{T}\right]}^{T}\in R^{4N+2}$ as input and produces a scalar state-action value $Q(s_{t},a_{t})$ as output. The higher input dimension of the critic reflects the need to evaluate how a specific combination of phase and position adjustments affects the beamforming performance. The two critics are initialized independently to provide diverse value estimates and reduce overestimation bias.

### 3.3. Training Procedure

The training procedure is summarized in Algorithm 1. Each episode starts from the center frequency-matched filter, i.e., $\delta_{m}^{\left(0\right)}=0$ and $\theta_{m}^{\left(0\right)}=∠\left({\left[h_{k_{c}}\left(\delta^{\left(0\right)}\right)\right]}_{m}\right)$ with $k_{c}=\left\lceil K/2\right\rceil$, and the agent refines the phase shifts and antenna positions over *T* steps. All network parameters are updated by the Adam optimizer with learning rate η. The deterministic policy is evaluated periodically, and the configuration with the highest minimum beamforming gain is kept as the final output.
**Algorithm 1** SAC-Based Joint Phase Shift and Antenna Position Optimization**Require:** System parameters *N*, *K*, $f_{c}$, *B*, $\delta_{B}$, user position $p_{u}$.

**Ensure:** Optimized $\theta^{*}$ and $\delta^{*}$.
 1: Initialize actor $\pi_{\phi }$, twin critics $Q_{\psi_{1}}$, $Q_{\psi_{2}}$, target networks $Q_{{\bar{\psi }}_{1}}$, $Q_{{\bar{\psi }}_{2}}$, replay buffer D.  
 2: **for** episode $=1,2,\ldots ,E_{\max}$ **do**
 3:     Initialize $\theta_{0}$ and $\delta_{0}$ via center frequency-matched filter.
 4:     Compute initial state $s_{0}$.
 5:     **for** t=0,1,…,T−1 **do**
 6:         Sample action $a_{t}∼\pi_{\phi }\left(\cdot |s_{t}\right)$.
 7:         Update $\theta_{t+1}$ and $\delta_{t+1}$ via (8)–(9).
 8:         Compute reward $r_{t}=\min_{k}G_{k}\left(\theta_{t+1},\delta_{t+1}\right)$.
 9:         Observe next state $s_{t+1}$.
10:        Store $\left(s_{t},a_{t},r_{t},s_{t+1}\right)$ in D.
11:        Sample mini-batch from D and update $\psi_{1}$, $\psi_{2}$, ϕ, $\alpha_{H}$.
12:        Soft-update target networks: ${\bar{\psi }}_{i}\leftarrow \tau \psi_{i}+\left(1-\tau \right){\bar{\psi }}_{i}$.
13:    **end for**
14: **end for** 
15: **return** $\theta^{*}$, $\delta^{*}$ with the highest $\min_{k}G_{k}$.


Note that the beamforming gains can be very small in magnitude, which makes direct use of raw gains in the state and reward numerically problematic for neural network training [26]. To address this, we apply a log-ratio transformation to the gain features. Let $G_{\min}^{\mathrm{ref}}$ and $G_{\mathrm{avg}}^{\mathrm{ref}}$ denote the minimum and average gains at the initial matched-filter configuration, respectively, i.e., $G_{\min}^{\mathrm{ref}}=\min_{k}G_{k}\left(\theta_{0},\delta_{0}\right)$ and $G_{\mathrm{avg}}^{\mathrm{ref}}=\frac{1}{K}\sum_{k}G_{k}\left(\theta_{0},\delta_{0}\right)$, where $\theta_{0}$ and $\delta_{0}$ are initialized as described above. The gain features in the state are computed as

(17) $${\tilde{G}}_{\min,t}=\ln\;\left(\frac{G_{\min,t}}{G_{\min}^{\mathrm{ref}}}\right),\;\;\;{\tilde{G}}_{\mathrm{avg},t}=\ln\;\left(\frac{G_{\mathrm{avg},t}}{G_{\mathrm{avg}}^{\mathrm{ref}}}\right),$$

and the phase shifts and antenna displacements are normalized to [−1,1] via $\theta_{t}/\pi$ and $\delta_{t}/\delta_{B}$, respectively. The reward is similarly transformed as

(18) $${\tilde{r}}_{t}=\ln\;\left(\frac{\min_{k}G_{k}\left(\theta_{t+1},\delta_{t+1}\right)}{G_{\min}^{\mathrm{ref}}}\right),$$

which maps the raw gain improvement into a logarithmic scale. This log-ratio reward provides meaningful gradient signals even when the absolute gain values are small, and a positive ${\tilde{r}}_{t}$ indicates that the current configuration improves over the initial matched-filter solution.

### 3.4. Discussion

**Figure 3:**
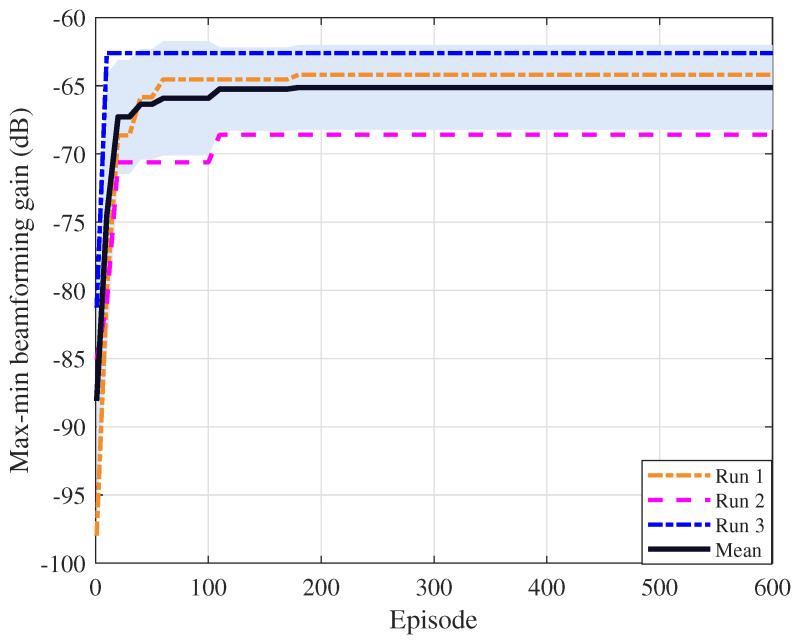
Convergence behavior of the proposed algorithm in terms of the max-min beamforming gain. The solid dark curve denotes the mean over three runs, and the shaded region represents the standard deviation across runs.

The proposed SAC-based algorithm follows the soft policy iteration framework [25]. In each training step, the actor and critic are updated using mini-batches sampled from the replay buffer, which can be viewed as a stochastic form of soft policy iteration. While exact convergence guarantees are available only in the tabular setting, experience replay, target networks, and twin critics are widely used to improve training stability. Figure 3 in Section 4.2 further demonstrates the reliable convergence behavior of the proposed algorithm.

The computational complexity of Algorithm 1 is analyzed as follows. At each training step, the dominant operations consist of channel gain computation and neural network updates. Evaluating the beamforming gain $G_{k}={\left|w^{H}h_{k}\right|}^{2}$ for all *K* subcarriers requires O(NK) operations, where *N* is the number of antennas. The actor network maps the state $s_{t}\in R^{2N+2}$ through two hidden layers of *H* neurons each and produces an action $a_{t}\in R^{2N}$; a single forward pass costs $O(NH+H^{2})$, and the backward pass is of the same order. Each of the twin critic networks takes the concatenated state-action pair ${\left[s_{t}^{T},a_{t}^{T}\right]}^{T}\in R^{4N+2}$ as input and outputs a scalar value, with a per-network cost of $O(NH+H^{2})$. Including replay-buffer sampling and target-network soft updates, the overall per-step training complexity is $O(NK+NH+H^{2})$. Over $E_{\max}$ episodes of *T* steps each, the total training complexity is $O(E_{\max}T\left(NK+NH+H^{2}\right))$. At deployment, only a single forward pass through the actor network is required, with an inference complexity of $O(NH+H^{2})$.

## 4. Simulation Results and Discussions

In this section, we present simulation results to evaluate the performance of the proposed SAC-based DRL algorithm for joint phase shift and antenna position optimization in a near-field wideband uplink system. Specifically, we first describe the simulation setup in Section 4.1, and then present the results in Section 4.2.

### 4.1. Simulation Setup

In our simulations, the BS is equipped with N=96 antenna elements aligned along the *y*-axis. The reference inter-element spacing is set to one wavelength at the carrier frequency $f_{c}=30$ GHz. The uplink user is located at $\left(x_{u},y_{u}\right)=\left(5\;m,5\;m\right)$ within the near-field region. Other key parameters are listed in Table 1. The bandwidth *B*, number of subcarriers *K*, and movable range $\delta_{B}$ vary across different simulations and are specified accordingly.

The proposed algorithm is compared with five baselines. The first is the per-subcarrier phase alignment scheme, which independently matches the combining vector to the channel at each subcarrier. It serves as a performance upper bound since it eliminates the beam split effect. The second is the DDPG algorithm, which uses the same MDP formulation but with a deterministic policy. The third is smoothed gradient descent-ascent (SGDA), which alternately updates the phase shifts and antenna positions via normalized gradient steps on a smooth approximation of the max-min objective. The fourth is phase-only optimization, which optimizes the analog phase shifts using projected gradient ascent on a smoothed max-min objective while keeping the antenna positions fixed. The fifth is fixed phase combining, which directly applies the center frequency-matched filter without any optimization.

### 4.2. Simulation Results

Figure 3 shows the convergence of the proposed algorithm with B=5 GHz, K=256, and $\delta_{B}=\lambda_{c}/2$. Three independent runs with different random seeds are conducted. The solid curve denotes the mean, and the shaded region represents the standard deviation. We can observe that all three runs converge to a similar level of the minimum beamforming gain, and the mean curve rises rapidly in the early phase and then levels off. Despite different random seeds, all runs achieve a clear improvement over the initial matched-filter configuration, which confirms the effectiveness and stability of the proposed algorithm.

Table 2 evaluates the sensitivity of the proposed algorithm to three key MDP design parameters, the phase step-size $\alpha_{\theta }$, the position step-size factor $\alpha_{\delta }$, and the number of steps per episode *T*. Each parameter is swept independently while fixing the others at their default values, with $\delta_{B}/\lambda_{c}=0.5$. As shown in Table 2, we can see that the proposed algorithm is robust to the choice of action step-sizes, with the minimum beamforming gain varying by less than 1.7 dB across the entire $\alpha_{\theta }$ range and less than 2.5 dB across the $\alpha_{\delta }$ range. Compared with the step-sizes, the episode length *T* affects the performance more noticeably. Specifically, when T=10, the agent has too few steps per episode to refine the configuration, and the minimum gain is only −73.4 dB. The best performance of −64.7 dB is achieved at T=50, beyond which no further improvement is observed as T=80 gives −66.9 dB. Thus, T=50 is adopted as the default value in the other simulations.

**Figure 4:**
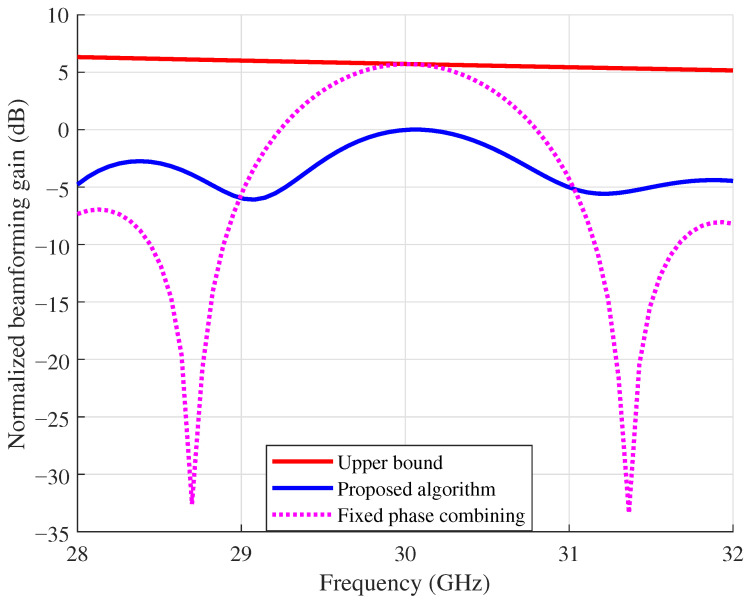
Normalized beamforming gain across subcarriers at B=4 GHz.

**Figure 5:**
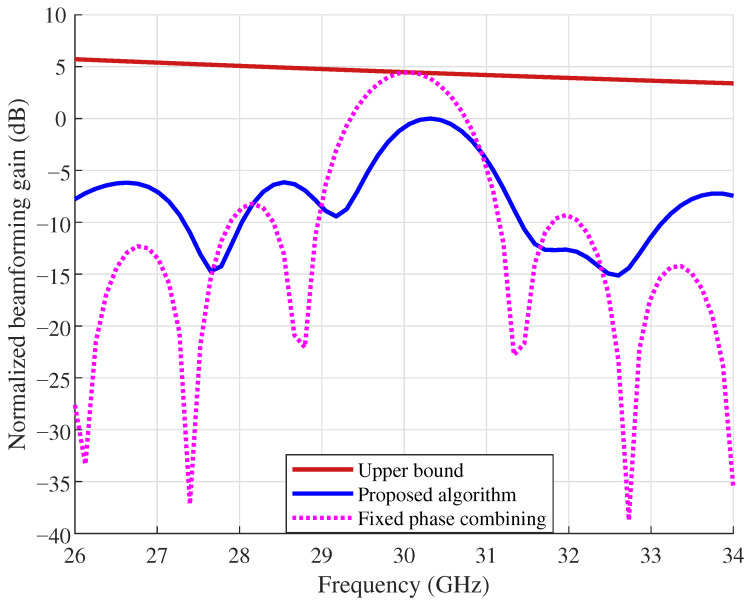
Normalized beamforming gain across subcarriers at B=8 GHz.

Figure 4 and Figure 5 compare the normalized beamforming gain across subcarriers for the proposed algorithm, the per-subcarrier upper bound, and fixed phase combining, under B=4 GHz and B=8 GHz with K=256 and $\delta_{B}=\lambda_{c}/2$, respectively. The normalized beamforming gain is obtained by dividing each per-subcarrier gain by the maximum per-subcarrier gain of the proposed algorithm, and is plotted in decibels. As shown in Figure 4, fixed phase combining suffers from deep nulls at certain subcarriers due to the beam split effect, whereas the proposed algorithm maintains a much flatter gain profile across the bandwidth. Although a gap to the upper bound remains, the gain variation in the proposed approach is significantly smaller than that of fixed phase combining. When the bandwidth increases to B=8 GHz in Figure 5, the beam split effect becomes more severe, and fixed phase combining exhibits deeper and more frequent nulls across the band. In contrast, the proposed algorithm still achieves a smoother gain distribution. Comparing the two figures, the advantage of the proposed approach over fixed phase combining is more pronounced at the larger bandwidth, which indicates that antenna position optimization is particularly effective when the beam split effect is strong.

**Figure 6:**
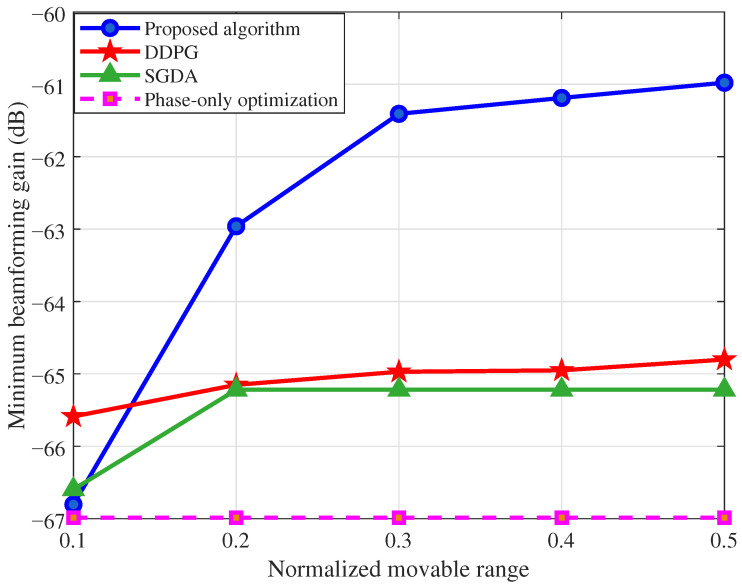
Minimum beamforming gain versus the normalized movable range $\delta_{B}/\lambda_{c}$.

Figure 6 shows the minimum beamforming gain as a function of the normalized movable range $\delta_{B}/\lambda_{c}\in \left\{0.1,\;0.2,\;0.3,\;0.4,\;0.5\right\}$ with B=5 GHz and K=256. The proposed SAC algorithm is compared with DDPG, SGDA, and phase-only optimization, and all results are averaged over ten independent random seeds. From Figure 6, we can see that the proposed SAC outperforms both DDPG and SGDA across all movable ranges except $\delta_{B}/\lambda_{c}=0.1$, where antenna movement is highly restricted and the gradient-based SGDA achieves comparable performance. As the movable range increases, SAC achieves a monotonically increasing minimum gain and demonstrates a clear advantage over the other methods. In contrast, DDPG and SGDA exhibit limited improvement with increasing movable range, as the enlarged search space and the non-smooth max-min structure cause the deterministic policy and gradient-based methods to converge to suboptimal local solutions. Phase-only optimization remains constant since it does not utilize antenna position adjustment. These results confirm that the proposed method is most beneficial where movable antennas offer sufficient spatial degrees of freedom.

## 5. Conclusions

This paper studied the beam split alleviation problem in a movable antenna-enabled near-field wideband uplink system with analog beamforming. A max-min optimization problem was formulated to jointly optimize the analog phase shifts and antenna positions. To solve this non-convex problem, an SAC-based DRL framework was developed, where the MDP was designed with the minimum beamforming gain as the reward and the phase/position adjustments as continuous actions. Simulation results showed that the proposed approach achieves a flatter beamforming gain distribution across subcarriers and consistently outperforms DDPG, SGDA, and phase-only optimization methods, especially when the antenna movable range is sufficiently large.

The present work focuses on a single-user near-field wideband MA array with analog beamforming under an LoS-dominant channel, and several extensions are worthy of pursuit in the future. First, as in [27,28], a two-timescale framework can be adopted, where the antenna positions are optimized on a large timescale based on statistical CSI and the phase shifts are updated on a small timescale with instantaneous CSI. Second, the robustness to imperfect CSI and multipath propagation can be studied by training over randomized channel realizations. Third, the joint phase-position design can be extended to multi-user or distributed MA-enabled near-field networks, where inter-user interference, coordination among movable arrays, and asynchronous information exchange need to be considered [29,30]. Finally, alternative reward formulations that explicitly penalize gain variance across subcarriers or balance minimum and average gains may further improve convergence speed and solution quality.

## Figures and Tables

**Table 1:** Simulation parameters.

Parameter	Value
Number of antenna elements *N*	96
Carrier frequency $f_{c}$	30 GHz
Reference inter-element spacing	$\lambda_{c}$
User location $\left(x_{u},y_{u}\right)$	(5 m, 5 m)
Discount factor γ	0.99
Soft update coefficient τ	0.005
Learning rate η	$3\times 10^{-4}$
Replay buffer size	$10^{5}$
Mini-batch size	256
Steps per episode *T*	50
Total episodes $E_{\max}$	2000
Phase step-size $\alpha_{\theta }$	0.02
Position step-size $\alpha_{\delta }$	$0.04\times \delta_{B}$
Hidden layer size	256

**Table 2:** Parameter sensitivity analysis.

Parameter	Swept Values				
$\alpha_{\theta }$	0.005	0.01	0.02	0.04	0.08
Min. gain (dB)	−67.5	−68.1	−67.8	−67.1	−66.4
$\alpha_{\delta }$	0.005	0.01	0.02	0.04	0.08
Min. gain (dB)	−68.3	−67.7	−69.1	−66.8	−68.2
*T*	10	20	30	50	80
Min. gain (dB)	−73.4	−70.4	−69.2	−64.7	−66.9

## Data Availability

Data is contained within the article.
